# Virological failure among adolescents on ART, Harare City, 2017- a case-control study

**DOI:** 10.1186/s12879-018-3372-6

**Published:** 2018-09-18

**Authors:** Zvanaka Sithole, Elizabeth Mbizvo, Prosper Chonzi, More Mungati, Tsitsi Patience Juru, Gerald Shambira, Notion Tafara Gombe, Mufuta Tshimanga

**Affiliations:** 1 MPH Programme, Department of Community Medicine, University of Zimbabwe, Harare, Zimbabwe; 2 City Health Directorate, Harare, Zimbabwe; 3 Elizabeth Glaser Pediatirc AIDS Foundation, Lesotho, Swaziland

**Keywords:** Viral load suppression, Adolescents, Case control, Harare city

## Abstract

**Background:**

Zimbabwe is on track towards achieving viral suppression among adults (87%). However, adolescents have only achieved 44% by 2016. In Harare city, 57% of adolescents had attained viral suppression after 12 months on ART compared to 88% among adults. We determined factors associated with virological failure among adolescents (age 10–19 years) on antiretroviral therapy (ART) in Harare city.

**Methods:**

We conducted a one to one unmatched case control study among 102 randomly recruited case: control pairs at the two main infectious disease hospitals in Harare. A case was any adolescent who presented with VL > 1000c/ml after at least 12 months on ART. A control was any adolescent who presented with VL < 1000c/ml after at least 12 months on ART. Interviewer administered questionnaires were used to collect data. Epi Info 7 was used to generate frequencies, means, proportions, ORs and *p*-values at 95% CI.

**Results:**

We interviewed 102 case-control pairs. Poor adherence to ART [aOR = 8.15, 95% CI (2.80–11.70)], taking alcohol [aOR = 8.46, 95% CI (3.22–22.22)] and non- disclosure of HIV status [aOR = 4.56, 95% CI (2.20–9.46)] were independent risk factors for virological failure. Always using a condom [aOR = 0.04, 95% CI (0.01–0.35)], being on second line treatment [aOR = 0.04, 95% CI (0.23–0.81)] and belonging to a support group [aOR = 0.41, 95% CI (0.21–0.80)] were protective.

**Conclusion:**

Poor adherence, alcohol consumption and non-disclosure increased the odds of virological failure. Based on these findings support should focus on behavior change and strengthening of peer to peer projects to help address issues related to disclosure and adherence. Further operational research should aim to define other components of effective adherence support for adolescents with virological failure.

## Background

Virological monitoring through plasma viral load (PVL) quantification is an essential part of clinical monitoring of HIV patients undergoing antiretroviral treatment therapy (ART), and for detecting treatment failure. In addition to showing how effective the ART is in suppressing the virus, it gives room for treatment to be modified to suite the strain of the virus in one’s body [1]. HIV viral load testing may also be used to help determine whether the virus infecting a person has become drug-resistant [2]. Virological failure and absence of clinical features of improvement often result from the development of resistant mutations necessitating a change of ART regimen [3].

The World Health Organization (WHO) recommends routine annual viral load (VL) monitoring for all patients on ART, as the most accurate available measure of treatment response [4]. This is also done as a strategy for the surveillance and monitoring of HIV drug resistance [5]. While viral load has been seen as a tool to diagnose failure, the main benefit is to identify patients in need of intensive adherence counselling and intervene by providing it. This would help keep patients on first line ART regimen for long and prevent unnecessary switch to second and third line ART which is costly to manage especially in developing countries like Zimbabwe.

Virological failure is classified as a Plasma viral load above 1000 copies/ ml based on two consecutive viral load measurements after 3 months, with adherence support. If using dry blood spot technology, a viral load above 3000 copies/ml based on two consecutive viral load measurements after 3 months, with adherence support is also classified as virological failure [5]. The Zimbabwe national guidelines of 2013 recommends patients with viral load copies greater than 1000/ml after being on ART for at least 6 months to be classified as having virological failure [6].

Other studies have shown that comorbidities like having TB on ART, lower baseline weight and drug to drug reactions are associated with virological failure [7]. Being on WHO stage 3 or 4 and having a low CD4 at ART initiation were also associated with virological failure [8]. Virological failure can be due to many factors like treatment interruption, resistant strains carried from mother to child transmission (MTCT), as well as exposure of the adolescent to resistant strain through the individual’s own infection due to unprotected sexual inter course [9]. The behavior related factors associated with virological failure include having unprotected sexual intercourse with an HIV positive partner, substance abuse, having sex under the influence of alcohol and alcohol intake. Previous studies highlighted some difficulties with drug adherence among adolescents, associated with lack of caregiver support, fear of disclosure, fear of stigma and discrimination [10].Correlates of ART non adherence among adolescents has been shown to include alcohol and substance use [10].

In 2014, a new global strategy was launched with the aim to end the AIDS epidemic by 2030. The Joint United Nations Programme on HIV/AIDS (UNAIDS) and partners set ambitious global targets for the year 2020. These include diagnosing 90% of all people living with HIV (PLWHIV), initiating antiretroviral treatment (ART) for 90% of those diagnosed with HIV infection and achieving an undetectable viral load in 90% of those on ART [11]. In view of this, the Government of Zimbabwe developed the viral load scale-up plan 2015–2018. The plan focuses on achieving the third 90 by 2018.

In Zimbabwe, viral load testing remains a challenge due to lack of viral load testing machines at hospitals [12]. In Harare city VL testing is done to a new patient at 6 months and 12 months. Thereafter once per year on the anniversary of the month of initiation.

The National Aids Council (NAC) 2017 report revealed that about 35% of the estimated one million people on ARVs are on second line treatment. While having people living with HIV on second line treatment is proving to be expensive for Zimbabwe, quite a sizeable number are failing this line as well and are being moved to a third line regimen.

According to the Zimbabwe Populations Based HIV Impact Assessment (ZIMPHIA - 2016), 87% of those on ART (15–64 years old) are virally suppressed. However, nationally, viral load suppression remains a challenge among adolescents (10–19 years) with viral load suppression of 48.6% among adolescent females and 40.2% among adolescent males being reported [13]. Zimbabwe is on track as far as viral load suppression among adults is concerned, with VLS prevalence of 87%. Although adolescents’ VLS showed a challenge (44%) at national level [13], the data could not be generalized as it focused on all people living with HIV (whether on ART or not). Nevertheless, Harare, the capital city of Zimbabwe is not spared from this challenge. A preliminary review of its viral load data showed that only 57% of adolescents have attained viral suppression after 12 months on ART compared to 88% among adults. Generally adolescents have challenges in sexual health and self-care [14]. We therefore determined the factors associated with virological failure among adolescents on antiretroviral therapy (ART) in Harare city.

## Methods

### Study design

We conducted a 1:1 unmatched case control study among adolescents on ART in Harare city. A 102 case- control pair was recruited into the study. A case was an adolescent (10-19 years) from Harare city who presented with a viral load more than 1000 copies/ml after at least 12 months on ART in Harare city, from January 2017 to June 2017. A control was an adolescent (10–19 years) from Harare city who presented with viral load below 1000 copies/ml after at least 12 months on ART in Harare city, from January 2017 to June 2017.

### Sampling

Beatrice Road Infectious Diseases Hospital (BRIDH) and Wilkins hospital being the main infectious diseases’ hospitals that manage both first and second line clients on ART in Harare city, were purposively selected. The OI/ART VL tests register was used as the sampling frame. Proportional sampling was done in accordance with the number of adolescents that received VL tests at each facility from January 2017 to June 2017. The entire cohort had a total of 392 adolescents and all received their due VL tests. BRIDH had 200 adolescents while Wilkins had 192. Fifty two cases were randomly selected from BRIDH viral load test register into the study, and 50 cases were also randomly selected from Wilkins VL test register. Random selection of cases was done using computer statistical randomizer that generated 102 numbers randomly after each unit had been sequentially numbered on the sampling frame. Controls from the same hospitals and age range were randomly selected using computer generated numbers.

Five key informants were purposively selected according to their knowledge on HIV/AIDS programming, management and performance monitoring and evaluation.

### Data collection

We used the structured pre tested interviewer-administered questionnaires to collect data. The questionnaires were designed in English language then translated into Shona language which is the local language for this population. Review of the hospital medical records provided baseline pre-ART data before initiation of HIV treatment as well as current treatment and clinical status. Cases and controls were interviewed either on their review dates or when they would have come for their support group meetings which were done on Thursdays, after every 2 weeks. Those who did not belong to any support group and did not come for review during study time, were interviewed at their homes. Contact details were found in the VL tests registers. Adherence was objectively assessed by doing a physical pill count of dose units taken between the supply date and the review date. Good adherence was defined as adherence necessary to achieve full and durable viral suppression, thus taking (= or > 95%) of the pills. Non adherence was defined as taking < 95% of the pills.

An interview guide for key informants was used to elicit information on the Harare city’s HIV program. Key informants interviews were also used to triangulate data collected from the clients on health service related factors.

### Data analysis

Data were analysed using Epi Info 7 to generate frequencies, means proportions and odds ratios. Using 95% confidence interval, we performed a bivariate analysis to assess associations between different exposures and virological failure. A *p*-value of < 0.05 was deemed statistically significant in this analysis. Multivariate analysis by way of a stepwise forward logistic regression model was performed on significant variables that were found in bivariate analysis.

Permission to conduct the study was obtained from the Harare city ethical review board, Joint Research Ethical Committee (Ref: JREC/164/16), and Medical Research Council of Zimbabwe (Ref: MRCZ/ B/1314). Written informed consent was sought and obtained from study participants who were 18 years and above. For participants aged below 18 years assent was obtained and written informed consent was obtained from their parents or guardians. Confidentiality was assured and maintained through no use of names on questionnaires and keeping questionnaires under lock and key.

### Definition of variables

#### Outcome variable

The dependent variable (outcome of interest) was virological failure. It was defined as a plasma viral load of > 1000copies/ ml blood.

#### Independent variables

Independent variables included the demographic characteristics of the study participants. Some of these include the age (Number of completed years), marital status (married or not married), gender (male or female), alcohol intake (consumption of alcohol (yes/no)), residence was categorical (high/medium/low), caregiver HIV status (positive or negative), caregiver employment status (employed or not employed), disclosure status (disclosed HIV status or not), WHO staging (WHO stage at ART initiation at ART initiation), CD4 count (CD4 count at ART initiation at ART initiation), Correct and consistent use of condoms(reporting always using a condom correctly each time they had sexual intercourse or not always using a condom), chronic illness (having a chronic disease or not), second line treatment(being on a protease inhibitor plus 2 nucleoside analogues(NRTIs) or not), and adherence (poor< 95% or good adherence > 95%).

## Results

### Socio-demographic characteristics of study participants

The study recruited 102 case–control pairs giving us a recruitment rate of 100% (Table 1). The majority were females, single and students. Both cases and controls had a median age of 18 years. There were no significant differences between cases and controls on any of the demographic characteristics.

**Table 1:** Demographic Characteristics of Study Participants in Harare City, 2017

Variable	Category	Cases (%)	Controls (%)	*p* value
Sex	Female	54 (53)	63(62)	0.20
	Male	48(47)	39(38)	
Marital Status	Married	7(6)	8 (8)	0.80
	Single	95(94)	94(92)	
Residence	High	74 (72)	75 (73)	0.76
	Medium	22 (22)	22 (22)	0.78
	Low	6 (6)	5 (5)	Referent
Education	Primary	16(16)	15(15)	0.56
	Secondary	76(75)	73(72)	0.50
	Tertiary	10(9)	13(13)	Referent
Employment	Informal	7(7)	6(6)	0.42
	None	45(45)	27(26)	0.16
	Student	48(46)	65(64)	0.66
	Formal	2(2)	4(4)	Referent
Religion	Pentecostal	38 (34)	31(27)	0.69
	Apostolic	11(11)	9(9)	0.79
	Orthodox	21 (22)	32(33)	0.20
	None	32(33)	30(31)	Referent
Orphan Status	Double orphan	40(39)	26(26)	0.10
	Orphan mother	14(14)	8(8)	0.053
	Orphan father	23(22)	30(30)	0.82
	Not orphan	25(25)	38(36)	Referent
Living status	Orphanage home	47(46)	67(66)	0.38
	Other caregivers	45(44)	31(30)	0.07
	Living with partner	8(8)	3(3)	0.05
	Living with parents	2(2)	1(1)	Referent
Age in years	Median age	18(Q_1_ = 16; Q_3_ = 19)	18(Q_1_ = 16;Q_3_ = 19)	
Duration on ART	Median months	52(Q_1_ = 28; Q_3_ = 76)	41(Q_1_ = 23;Q_3_ = 60)	

### Socio-demographic factors associated with Virological failure among adolescents

Of the socio- demographic factors assessed, only two of the variables impacted on the outcome **(**Table 2). Those who had treatment buddies (OR = 0.4 CI = 0.16–0.96) were 0.6 times less likely to have virological failure as compared to those who did not. Those who had HIV positive care givers (OR = 0.50 CI = 0.28–0.88) were 0.5 times less likely to develop virological failure as compared to those who did not.

**Table 2:** Socio-demographic Factors Associated with Virological Failure among Adolescents on ART in Harare City, 2017

Variable	Category	Cases (%) (*n* = 102)	Controls (%) (*n* = 102)	OR	95%CI
Age	10-14 yrs	18(18)	24(24)	0.70	0.35–1.38
	15–19	84(82)	78(48)		
Sex	Female	54 (53)	63 (62)	0.70	0.40–1.22
	Male	48 (47)	39 (38)		
Having a treatment buddy	Yes	84(82)	94(92)	0.40	0.16–0.96*
	No	18(18)	8 (8)		
Family support	Yes	81(79)	86(84)	0.70	0.35–1.47
	No	21(21)	16(16)		
Support group involvement	Yes	27(26)	57(56)	0.30	0.16–0.51*
	No	75(74)	45(44)		
Caregiver not employed	Yes	8 (8)	3 (3)	2.81	0.72–10.91
	No	94(92)	99(97)		
Care giver HIV status	Positive	52(51)	69(68)	0.50	0.28–0.88*
	Negative	50(49)	33(32)		
Orphan status	Yes	77 (78)	65(64)	1.75	0.96–3.21
	No	25 (12)	37 (36)		

### Behavior related factors associated with Virological failure among adolescents

All except one of the factors assessed increased the odds of virological failure **(**Table 3). Significant behavior related risk factors associated with virological failure were having sex under the influence of alcohol (OR = 16.57, CI 3.34–82.28), Alcohol use (OR = 9.9, CI 3.96–24.76), smoking (OR = 9.3, CI 2.08–41.61), poor adherence (OR = 7.05, CI 3.80–13.08), not disclosing HIV status (OR = 5.88, CI 2.94–11.11), having more than one sexual partner for the previous 12 months (OR = 5.4, CI 1.57–18.57), busy schedules (OR = 4.32, CI 2.23–8.19), and being sexually active (OR = 2.74, CI 1.51–4.97). Correct and consistent use of a condom (OR = 0.11, CI 0.03–0.37) was a protective factor. There were no significant differences between female and male adolescents on condom use.

**Table 3:** Behavior Related Factors Associated with Virological Failure among Adolescents on ART in Harare City, 2017

Variable	Category	Cases	Controls	OR	95%CI
Alcohol use	Yes	39 (38)	6 (6)	9.9	3.96–24.76*
	No	63(62)	96 (94)		
Smoking	Yes	16(16)	2 (2)	9.3	2.08–41.61*
	No	86 (84)	100 (98)		
Substance abuse	Yes	9(9)	3(3)	3.2	0.80–12.16
	No	93(91)	99(97)		
Use of herbs	Yes	26(25)	8(8)	4.0	1.70–9.30*
	No	76(75)	94(92)		
Poor adherence	Yes	77 (75)	31 (30)	7.05	3.80–13.08*
	No	25 (25)	71 (70)		
Busy schedules	Yes	47 (46)	17 (17)	4.32	2.23–8.19*
	No	55 (54)	85 (83)		
Being sexually active	Yes	48 (47)	25 (25)	2.74	1.51–4.97*
	No	54 (53)	77 (75)		
> 1 sexual partner in the last 12mnth	Yes	24(55)	4 (18)	5.4	1.57–18.57*
	No	20 (45)	18 (82)		
Correct &consistent use of a condom	Always	6 (15)	13 (62)	0.11	0.03–0.37*
	Sometimes	34(85)	8 (38)		
Having sex under the influence of alcohol	Yes	29(67)	2 (11)	16.57	3.34–82.28*
	No	14 (33)	16(89)		
Not disclosing HIV status	Yes	51 (50)	15 (15)	5.88	2.94–11.11*
	No	51 (50)	87 (85)		

*Stastistically significant

### Treatment related factors associated with Virological failure among adolescents, Harare City, 2017

Those who started ART with CD4 < 350 cells/μl (OR = 4.76; CI 2.43–9.33) were five times more likely to develop virological failure as compared to those who started ART with CD4 > 350 cells/μl (Table 4). Those who started ART on WHO stages 3 or 4 of the disease (OR = 3.13, CI 1.76–5.55) were three times more likely to have virological failure as compared to those who started ART on WHO stages 1 or 2. Adolescents who had a chronic illness (OR = 2.76; CI 1.02–7.42) were almost three times more likely to develop virological failure as compared to those who were not. Adolescents who were on other medications apart from ART (OR = 1.48, CI 0.54–4.04) were 1.5 times more likely to develop virological failure as compared to those who were on ART only.

**Table 4:** Treatment Related Factors Associated with Virological Failure among Adolescents on ART in Harare City, 2017

Variable	Category	Cases (%)	Controls (%)	OR	95%CI
Being on other medication	Yes	10 (10)	7 (7)	1.48	0.54–4.04
	No	92 (90)	95 (93)		
WHO staging	3&4	60 (59)	32 (33)	3.13	1.76–5.55
	1&2	42 (41)	70 (67)		
CD4 count	≤350	87 (85)	56 (55)	4.76	2.43–9.33
	> 350	15 (15)	46 (45)		
Duration on ART	≤18 months	84(82)	86(84)	0.87	0.41–1.82
	> 18 months	18(18)	16(16)		
Chronic illness	Yes	15 (15)	6 (6)	2.76	1.02–7.42
	No	87(15)	96 (92)		
Drug S/E	Yes	18 (18)	12 (12)	1.61	0.73–3.53
	No	84 (82)	90 (88)		
Having no history of OIs	Yes	63(62)	70(69)	0.67	0.38–1.21
	No	39(38)	32(31)		
TDF + 3TC + EFV	Yes	19(19)	38(37)	0.34	0.20–0.73
	No	83(81)	64(63)		
Being on 2nd line treatment	Yes	26 (25)	5 (5)	0.15	0.06–0.4
	No	76 (75)	97 (95)		

Those on 2nd line treatment (OR = 0.15; CI 0.06–0.41) were less likely to have virological failure as compared to the first liners. Adolescents on ART for less than 18 months were less likely to develop virological failure compared to those with more than 18 months on ART (OR = 0.87; CI 0.41–1.82). ART regimen was also associated with virological failure. Being on TDF +3TC + EFV was protective.

### Independent factors associated with Virological failure among adolescents on ART, Harare City, 2017

Multivariate analysis by way of a stepwise forward logistic regression model was performed on significant variables that were found in bivariate analysis (Table 5). Poor adherence to ART (aOR = 18.15, CI 2.80–18.70), taking alcohol (aOR = 8.46, CI 3.22–22.22) and non- disclosure (aOR = 4.56, CI 2.20–9.46) were the independent risk factors. The independent protective factors were correct and consistent use of a condom (aOR = 0.4, CI 0.01–0.35, being on second line treatment (aOR = 0.04, CI 0.01–0.35) and having a support group (aOR = 0.41, CI 0.21–0.80).

**Table 5:** Independent Factors Associated with Virological Failure among Adolescents on ART, Harare City, 2017

Variable	Crude OR	aOR	95%CI
Poor adherence	7.05	8.55	2.80–11.70
Alcohol consumption	9.9	8.46	3.22–22.22
Non-disclosure	5.88	4.56	2.20–9.46
Having a support group	0.33	0.41	0.21–0.80
Consistently and correctly using a condom	0.11	0.04	0.01–0.35
Being on second line treatment	0.15	0.04	0.23–0.81

## Discussion

The study investigated the factors associated with virological failure among adolescents on ART and revealed that poor adherence to treatment, taking alcohol and non-disclosure of HIV status increased the odds of virological failure. The less likelihood factors included having a support group and being on 2nd line treatment. Correct and consistent use of a condom was a protective factor.

The study demonstrated that adolescents who were not adhering to ART were less likely to achieve viral suppression as compared to those who were adhering to ART. This is biologically plausible as poor adherence result in drug dosages below the level necessary to produce a therapeutic effect, thereby enabling development of drug resistance [15]. Our results are in line with those of Evans et al., 2013 in South Africa suggesting that poor adherence is associated with a higher risk of virological failure [16, 17]. The benefits of a combination therapy like Tenolam E (a 3 drug regimen -Tenofovir + Lamivudine+ Efavirenz) in this study could be due to its once-daily regimen which has less pill burden as compared to other regimens.

In this study, adolescents who reported being on other medications apart from ART were more likely to develop virological failure as compared to those who were on ART only. Although the study did not assess why adolescents on other medications were more likely to develop virological failure, this could be due to several explanations like by the pill burden that would be encountered by adolescents that would be on other medications. Other studies demonstrated that pill burden among adolescents is associated with poor adherence [18]. This could possibly be as a result of drug to drug interaction or the disease severity of those on other medications.

The odds of virological failure increased with disease severity at ART initiation. Virologic failure was more likely in advanced disease (WHO stage 3 and 4, CD4 < 350 cells/mm^3^). This shows delay in ART initiation among adolescents. Chimbetete et al., 2011 also found that patients initiated on ART after developing AIDS related illness (WHO stage 3 & 4), and those who had low baseline CD4 counts had higher risk of treatment failure as compared to those in WHO stage 1 or 2 and those with high CD4 [8]. However the 2015 WHO guidelines expanded ART initiation to treat all. This will minimize the delays on ART initiation but delayed diagnosis would remain a significant problem. Similar findings from other studies suggest that adolescents who delay ART initiation are more likely to have resistant mutations [19, 20].

The independent association of alcohol consumption and virological failure is not surprising since alcohol use among adolescents has been associated with lower treatment adherence, disease progression and failed viral suppression. One study by Matare et al., 2014 examined the association of alcohol with viral suppression and found that those who consumed alcohol were more likely to develop virological failure compared to those who did not [7]. In another study by Adrovandi et al., 2014, alcohol use was associated with poor adherence [21]. In contrast, Chimbetete et al., 2011 found no difference in treatment failure among those who were alcohol drinkers [8]. This could be explained by the levels of drinking alcohol in that study, which had no effect on their drug adherence levels.

Disclosure has been shown to be a protective factor as far as viral suppression is concerned. This could be due to the association between disclosure of HIV status and adherence. Some studies have found delayed or nondisclosure of HIV status to be associated with virological failure [22]. Attention should be focused on increased involvement of the child and family in medical treatment after disclosure. Disclosed children have better access to social support and tend to be less depressed over the long-term, thereby adhering to their medication well [22].

In this study, belonging to a support group was a less likelihood factor. Ayer et al., 2016 also found that belonging to a support group was associated with viral suppression [23]. Our study also demonstrated that older adolescents were more likely to develop virological failure as compared to the younger adolescents. This is consistent with Cowan et al., 2009 on viral load suppression among adolescents in sub-Saharan Africa, who also found that older adolescents were more likely to have virological failure as compared to the younger adolescents [24]. The increased risk of virological failure among older adolescents could be explained by the developmental milestones gone through by older adolescents in transition to their adulthood as compared to the younger adolescents. These include social, emotional and cognitive changes [25].

Those who were on second line treatment were less likely to develop virological failure as compared to those on first line treatment regimen. However, the additional counselling among adolescents on second-line treatment might have somewhat biased this study finding. Those who are on second line treatment receive enhanced adherence unlike those on first line treatment.

The strengths of this study included the triangulation of information given by the study participants with the information included in the clinical records. However, our study had some limitations. The Information provided on exposure differed between cases and controls. Cases could accurately recall past exposures better than controls resulting in recall bias. However some of the given information were verified with medical records. Some of the questions included in the questionnaires were sensitive and this could have introduced social desirability bias. Also adherence was assessed using pill count method which not a perfect method of assessing adherence. Despite these shortcomings, this study fills a gap in research on factors associated with virological failure in Harare city, Zimbabwe.

## Conclusion

The study demonstrated that poor adherence was strongly associated with virological failure among adolescents on ART in Harare city. Although consistent and correct use of condoms, second line ART regimens and belonging to a support group were protective against virological failure, taking alcohol and non-disclosure also increased the odds of virological failure. Following this, findings of this study were used by Harare city to lobby for supply of more ARV drugs to maintain the 3 months minimum stock from the ministry of health. The program manager also used these findings to improve on adolescent friendly services through rehabilitation of one of the two non-functional adolescent treatment corners that were in place. An awareness campaign on ART adherence and behavioral change was also conducted. Based on these findings support should focus on behavior change and strengthening of peer to peer projects to help address issues related to disclosure and adherence. Further operational research should aim to define other components of effective adherence support for adolescents with virological failure.
